# Celecoxib Improves Host Defense through Prostaglandin Inhibition during *Histoplasma capsulatum* Infection

**DOI:** 10.1155/2013/950981

**Published:** 2013-04-22

**Authors:** Priscilla Aparecida Tartari Pereira, Bruno Caetano Trindade, Adriana Secatto, Roberto Nicolete, Camila Peres-Buzalaf, Simone Gusmão Ramos, Ruxana Sadikot, Claudia da Silva Bitencourt, Lúcia Helena Faccioli

**Affiliations:** ^1^Departamento de Análises Clínicas, Toxicológicas e Bromatológicas, Faculdade de Ciências Farmacêuticas de Ribeirão Preto, Universidade de São Paulo, Avenida do Café, s/n, 14040-903 Ribeirão Preto, SP, Brazil; ^2^Departamento de Patologia, Faculdade de Medicina de Ribeirão Preto, Universidade de São Paulo, 14049-900 Ribeirão Preto, SP, Brazil; ^3^University of Florida, Gainesville, FL 32669, USA

## Abstract

Prostaglandins act as mediators of inflammation and, similar to cytokines, function as immune modulators during innate and adaptive immune responses. Therefore, using a pharmacological inhibitor, celecoxib, we investigated the role of prostaglandins in host defense against *Histoplasma capsulatum* infection in C57BL/6 mice. Our results showed that treatment with celecoxib inhibited cyclooxygenase 2, reduced the total fungal burden, and reduced the concentration of PGE_2_, cytokines, lymphocytes, neutrophils, and mononuclear cells in the bronchoalveolar space and lung parenchyma. In addition, celecoxib treatment increased the synthesis of nitric oxide, IFN-**γ**, LTB_4_, and the phagocytic capacity of alveolar macrophages. Moreover, celecoxib treatment increased the survival of mice after infection with a lethal inoculum of *H. capsulatum*. These results suggest that prostaglandins alter the host immune response and play an important role in the pathogenesis of histoplasmosis. Thus, the inhibition of prostaglandins could be a valuable immunomodulatory strategy and antifungal therapy for histoplasmosis treatment.

## 1. Introduction

*Histoplasma capsulatum* is a dimorphic fungus that develops a yeast-like morphology in host tissue that leads to the development of pulmonary disease characterized by chronic granulomatous and suppurative inflammatory reactions [1, 2] and the increased expression of cytokines, including interleukin- (IL-) 12, interferon- (IFN-) *γ*, tumor necrosis factor- (TNF-) *α*, granulocyte macrophage colony-stimulating factor (GM-CSF), IL-17, and IL-23, which are critical for the immunoprotective response in *H. capsulatum*-infected mice [3–7]. We have previously demonstrated that leukotrienes are important for efficient host defense against *H. capsulatum* infection [8]; however, the role of prostaglandins has not been studied. Prostaglandins (PGs) are synthesized through the actions of phospholipase A_2_ (PLA_2_) and cyclooxygenases (COXs) on the arachidonic acid (AA) released from cellular membrane phospholipids [9, 10]. COXs exist as two major isoforms: COX-1 and COX-2. COX-1 is the enzyme responsible for basal and constitutive prostaglandin synthesis, and COX-2 is important in various inflammatory responses [11]. COX-2 is induced through a variety of stimuli received from *in vitro* and *in vivo* proinflammatory environments [12, 13]. COX-1 and COX-2 generate prostaglandins, such as PGI_2_, PGF_2*α*_, PGD_2_, and PGE_2_, with a wide range of actions [14, 15]. PGE_2_ exerts an immunosuppressive effect, inhibiting human T lymphocyte activation and proliferation and reducing the synthesis of Th1 cytokines such as IL-2 and IFN-*γ*. PGE_2_ also enhances IL-10, IL-4, and IL-5 production to induce the Th2 response [16, 17]. Moreover, PGE_2_ inhibits macrophage host defense functions such as phagocytosis and killing against bacterial infections [18, 19].

 The inhibition of COX-2 with agents such as celecoxib is widely used in clinical conditions for the treatment of a variety of inflammatory disorders [10, 17]. These inhibitors were designed based on the hypothesis that selective inhibition of the COX-2 isoform reduces pain and inflammation without compromising the integrity of the gastric mucosa [10, 17]. Although the role of COX-2-derived lipid mediators in inflammation is well known, their role in the immune response and host defense is not well defined. Therefore, we used celecoxib, a selective COX-2 inhibitor, to characterize the role of prostaglandins in host defense during experimental *H. capsulatum* infection. Here, we show that celecoxib treatment significantly reduces the fungal burden in the lungs and spleen of mice infected with *H. capsulatum*. This protective effect was mediated through an increase in the production of nitric oxide, leukotriene (LT)B_4_, and IFN-*γ*, followed by an enhancement of the phagocytic index and activation of macrophages. Furthermore, we show that mice infected with a lethal inoculum of *H. capsulatum* showed increased survival when treated daily with celecoxib. To our knowledge, this report is the first to demonstrate that celecoxib improves the host immune response against *H. capsulatum*, and prostaglandins play a key role in the immunopathogenesis of *H. capsulatum* infections. Therefore, we suggest that the inhibition of COX-2 could be an important adjuvant therapy for the treatment of histoplasmosis.

## 2. Materials and Methods

### 2.1. Animals

Specific pathogen-free male 6-week-old C57BL/6 mice (20–22 g) were obtained from the animal facilities of the Faculdade de Ciências Farmacêuticas de Ribeirão Preto, Universidade de São Paulo. All experiments were approved and conducted in accordance with guidelines of the Animal Care Committee of Universidade de São Paulo (protocol 07.1.365.53.8). The infected animals were maintained in biohazard facilities and housed in cages within a laminar flow safety enclosure under standard conditions.

### 2.2. Preparation of *H. capsulatum*

The strain of *H. capsulatum *was isolated from a patient at the Hospital das Clínicas, Faculdade de Medicina de Ribeirão Preto, Universidade de São Paulo. The fungi were cultured to the mycelial phase at 25°C in Sabouraud dextrose agar tubes (Difco, Detroit, MI), and the live yeast fungus was subcultured at 37°C on 5% glucose-cysteine-blood sheep brain heart infusion (BHI) agar (Difco) for 15 days. The yeast cultures were used at ≥90% viability according to fluorescein diacetate (Sigma-Aldrich, St. Louis, MO) and ethidium bromide (Sigma-Aldrich) staining [8].

### 2.3. Infection of Mice with *H. capsulatum*

The mice were anesthetized with 2.5% tribromoethanol and restrained on a small board. A 30-gauge needle attached to a tuberculin syringe was inserted into the trachea, and 100 *μ*L of phosphate-buffered saline (PBS) containing a lethal inoculum (1 × 10^6^ yeast/100 *μ*L/mouse) or sublethal inoculum (5 × 10^5^ yeast/ 100 *μ*L/mouse) of *H. capsulatum* was intratracheally (i.t.) dispensed into the lungs. The appropriate inoculum size was determined using a dose-response survival curve (data not shown).

### 2.4. Treatment of Mice with Celecoxib

The animals were divided into four groups. Group 1 mice were infected i.t. with *H. capsulatum *and treated with water (0.5 mL p.o. gavage) at 1 h before infection and daily for 30 days. Group 2 mice were infected i.t. with* H. capsulatum *and treated with celecoxib p.o. (1 mg/kg/0.5 mL) at 1 h before infection and daily for 30 days. Group 3 mice were inoculated i.t. with PBS (100 *μ*L) and treated with water p.o. (0.5 mL) daily for 30 days. Group 4 mice were injected i.t. with PBS and treated p.o. with celecoxib (1 mg/kg/0.5 mL) daily for 30 days.

### 2.5. Bronchoalveolar Lavage Fluid (BALF)

At 2, 14, and 28 days after infection, the mice were euthanized with CO_2_, and the BALF was collected as previously described [8]. The total cell counts were determined using a Neubauer chamber. The differential cell counts were obtained using a cytospin preparation stained with Panoptic (Laborclin, Paraná, Brazil).

### 2.6. Phagocytic Index (PI)

The total number of intracellular *H. capsulatum* yeasts in alveolar macrophages from the BALF and the number of mouse cells infected with at least one yeast of *H. capsulatum* were counted using a light microscope at a 100x magnification. The PI was calculated using the following equation: PI = number of engulfed yeasts × number of alveolar macrophages containing at least one yeast/total number of alveolar macrophages counted (200 mononuclear cells). 

### 2.7. Determination of Colony-Forming Units (CFU)

The recovery of *H. capsulatum *yeasts from the lung and spleen was performed as previously described [8]. Two-hundred-microliter samples from each cell suspension were collected, and a 10-fold dilution was plated onto BHI blood agar. After incubation at 37°C for 21 days, the *H. capsulatum *CFU were counted. The results are expressed as CFU/g lung or CFU/spleen.

### 2.8. Measurement of PGE_2_, LTB_4_, and Cytokines

The lungs were removed at 2, 14, or 28 days after infection, weighed, and homogenized (Mixer Homogenizer; Labortechnik, Staufen, Germany) in 1 mL of RPMI 1640 (10 mM indomethacin/1 mM EDTA). After centrifugation at 400 *g*, the supernatants were filtered and stored at −70°C until further analysis. The lipids were purified from filtered lung homogenate supernatants using Sep-Pak C_18_ cartridges according to the manufacturer's instructions (Waters Corp., Milford, MA). The quantification of PGE_2_ and LTB_4_ was performed using a specific enzyme immunoassay kit (Cayman Chemical, Ann Arbor, MI) according to the instructions of the manufacturer, and the results are expressed in ng/mL. Commercially available enzyme-linked immunosorbent assay (ELISA) antibodies were used to measure TNF-*α*, IL-1, IL-6, IL-10, IL-12, keratinocyte-derived chemokine (KC), and IFN-*γ* levels (R & D Systems, Minneapolis, MN).

### 2.9. Quantitation of Nitric Oxide (NO)

The amount of nitrite (NO_2_^−^) in lung homogenates was measured using the Griess reaction to determine the NO production, as previously described [20]. The values were determined using a standard curve with serial dilutions of NaNO_2_ (Sigma-Aldrich, St. Louis, MO).

### 2.10. Histology

The lungs were collected at 2, 14, or 28 days after infection and immediately fixed in 10% formalin. The specimen was processed, embedded in paraffin, and cut into 5 *μ*m sections. The sections were stained with hematoxylin and eosin (H & E) or Grocott's methenamine silver (GMS). The sections were analyzed in a blinded fashion.

### 2.11. Immunophenotyping of Lung Parenchyma Cells Using Flow Cytometry

The CD11c, MAC-3, GR-1, CD4, and CD8 expression was determined using the flow cytometry immunostaining protocol with antibodies conjugated to fluorochromes (BD Biosciences, NJ, USA). The lung parenchyma cells were adjusted to 1 × 10^6^ cells/mL. Subsequently, the cells were washed with PBS containing 2% fetal bovine serum (FBS), centrifuged at 400 *g*, and fixed with PBS containing 1% (w/v) paraformaldehyde. A total of 10,000 events were acquired for each tube (FACSCanto TM; Becton Dickinson, CA, USA), using the FACSDiva software. The numbers of CD4 and CD8-positive cells from the lung parenchyma were assessed through a specific gate for lymphocyte populations. A side-scatter gate was used to assess the myeloid populations using CD11c, MAC-3, and GR-1 markers. 

### 2.12. Statistical Analysis

The statistical analysis was performed using GraphPad software (San Diego, CA, USA). The data are expressed as the means ± standard error of the mean. For comparisons among groups, we used analysis of variance (ANOVA). The survival analyses were performed using the logrank test. *P* values < 0.05 were considered significant. 

## 3. Results

### 3.1. Celecoxib, a COX-2 Inhibitor, Reduces the Fungal Burden in the Lungs and Spleen of Mice Infected with *H. capsulatum*

**Figure 1:**
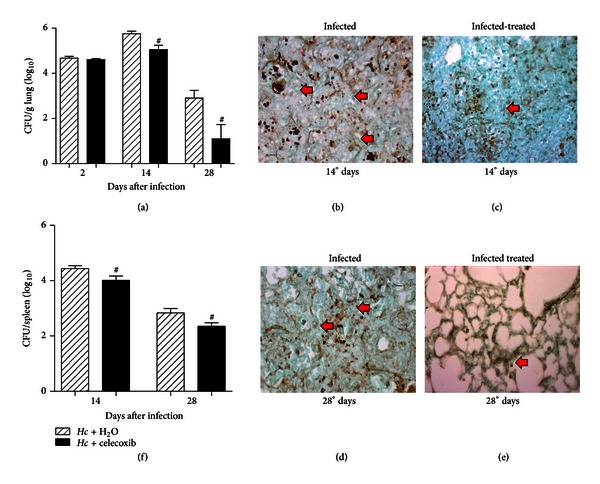
Celecoxib reduces the fungal burden in the lungs and spleen of *H. capsulatum*-infected mice. The fungal burden in the lungs (a) and spleen (f). Tissue samples were harvested at 2, 14, or 28 days after the sublethal inoculation of *H. capsulatum*. At 14 (b, c) and 28 (d, e) days after infection, the lung sections from mice receiving daily water (b, d) or celecoxib treatment (c, e) were stained with Grocott's methenamine silver (GMS) to detect yeast cells (black arrow shows the *H. capsulatum *yeast). Original magnification: 360x. The data are presented as the means ± SEM representative of three independent experiments (*n* = 5-6). One-way ANOVA (Tukey's multiple comparison test) was used. ^#^ *Hc* + H_2_O versus *Hc* + celecoxib *P* < 0.05.

Mice infected with a sublethal inoculum of *H. capsulatum *and treated with 1 mg/kg of celecoxib showed a significant reduction of the fungal burden in the lungs after 14 and 28 days at 0.84log⁡_10_ and 1.81log⁡_10_, respectively (Figure 1(a)). The fungal burden in the spleen was also reduced after celecoxib treatment at 0.42log⁡_10_ and 0.48log⁡_10_ after 14 and 28 days, respectively, compared with water-oral-treated infected mice (Figure 1(f)). To detect the presence of *H. capsulatum* yeast in the lung parenchyma, histological sections were generated and analyzed, which showed an increase of yeast in the lung parenchyma at 14 days (Figure 1(b)), and this increase was subsequently reduced after celecoxib treatment (Figures 1(c) and 1(e)).

### 3.2. The Inhibition of Prostaglandins Increases Macrophage-Mediated Phagocytosis of *H. capsulatum* Yeast and NO Production in the Lung Parenchyma

**Figure 2:**
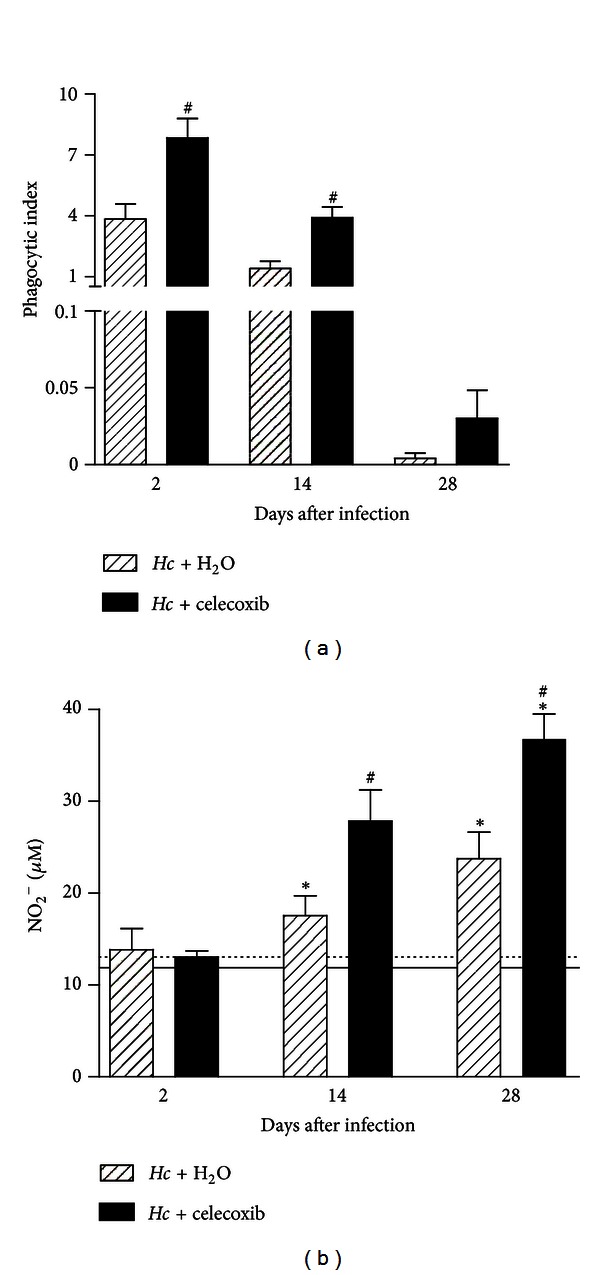
Celecoxib treatment increases the phagocytosis of yeast by alveolar macrophages *in vivo* and NO production in the lung parenchyma. (a) The BALF cells from mice infected with a sublethal inoculum of *H. capsulatum*, with or without celecoxib treatment, were collected and stained after cytospin preparation. The yeast cells in the alveolar macrophages were counted using bright-field microscopy at 100x magnification. The phagocytic index was calculated as described in Section 2. (b) The NO_2_^−^ production in the lung parenchyma was measured using Griess reagent. The data are presented as the means ± SEM representative of three independent experiments (*n* = 5-6). One-way ANOVA (Tukey's multiple comparison test) was used. *PBS + H_2_O versus *Hc* + H_2_O or versus *Hc* + celecoxib; ^#^ *Hc* + H_2_O versus *Hc* + celecoxib *P* < 0.05.

To determine the mechanism underlying the celecoxib-mediated reduction of the yeast burden, we investigated the effects of prostaglandin inhibition on the phagocytosis of *H. capsulatum* yeasts. BALF cells from infected mice treated with celecoxib (1 mg/kg p.o.) or vehicle (water) were recovered at 2 and 14 days after infection, and the number of intracellular yeasts in alveolar macrophages was determined. The cells recovered from celecoxib-treated infected mice exhibited greater fungal uptake than those obtained from mice treated with vehicle (Figure 2(a)). In addition, the treatment of infected mice with celecoxib resulted in a significant increase of NO production in lung parenchymal cells at 14 (~35%) and 28 days after infection (~55%) compared with mice treated with vehicle (Figure 2(b)). 

### 3.3. Celecoxib Inhibits PGE_2_ Production but Increases the Synthesis of LTB_4_ after *H. capsulatum* Infection

**Figure 3:**
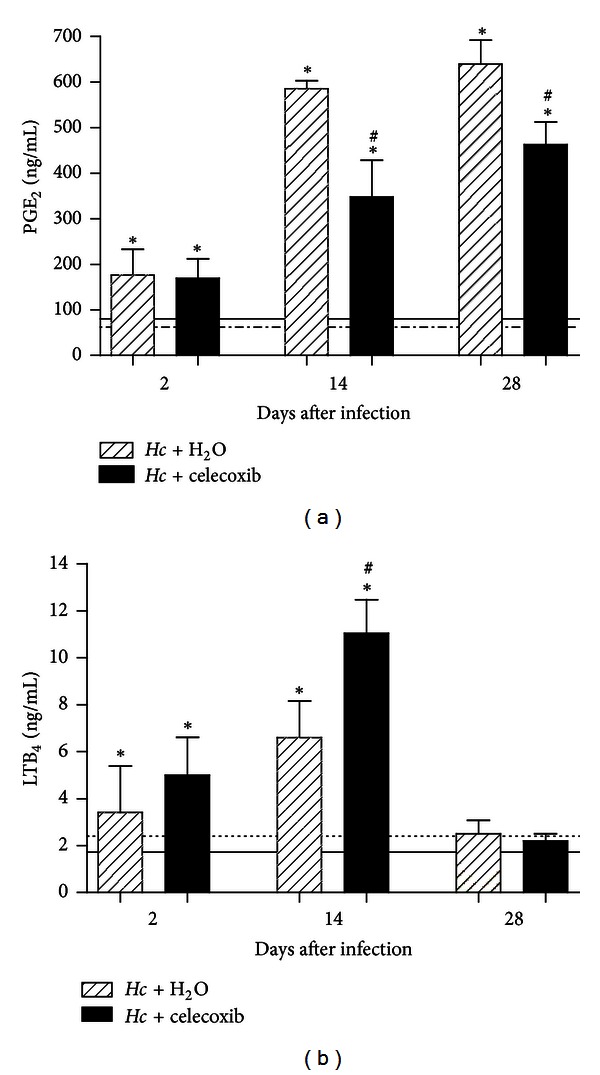
Celecoxib treatment modulates endogenous PGE_2_ and LTB_4_ production in the lungs of *H. capsulatum*-infected mice. Lipid mediators in the lung parenchyma were measured using enzyme immunoassay (a) PGE2, (b) LTB4. The lungs of mice infected i.t. with sublethal *H. capsulatum* inoculum and daily p.o. treatment with water or celecoxib (1 mg/kg/0.5 mL) were removed at 2, 14, or 28 days after infection and homogenized in medium in the presence of indomethacin. A group of noninfected mice receiving water (solid line) and noninfected mice treated with celecoxib (dotted line) were used as controls. The data are presented as the means ± SEM representative of three independent experiments (*n* = 5-6). One-way ANOVA (Tukey's multiple comparison test) was used. *PBS + H_2_O versus *Hc* + H_2_O or versus *Hc* + celecoxib; ^#^ *Hc* + H_2_O versus *Hc* + celecoxib *P* < 0.05.

We also examined the kinetics of PGE_2_ and LTB_4_ production during *H. capsulatum* infection and the impact of celecoxib on the concentration of these lipid mediators. We measured both lipids in lung homogenate samples from all experimental groups. The sublethal inoculum of *H. capsulatum *significantly induced PGE_2_ production during infection at 28 days after infection (Figure 3(a)). The celecoxib-treated uninfected control mice showed low levels of PGE_2_ during the entire experimental period. Infected mice treated with celecoxib showed significantly reduced PGE_2_ production at 14 and 28 days after infection. However, celecoxib induced the increased production of LTB_4_ (~56%) at 14 days after infection (Figure 3(b)), compared with infected mice without celecoxib treatment.

### 3.4. The Leukocyte Recruitment in the Lungs Was Reduced in Infected Mice Treated with Celecoxib

**Figure 4:**
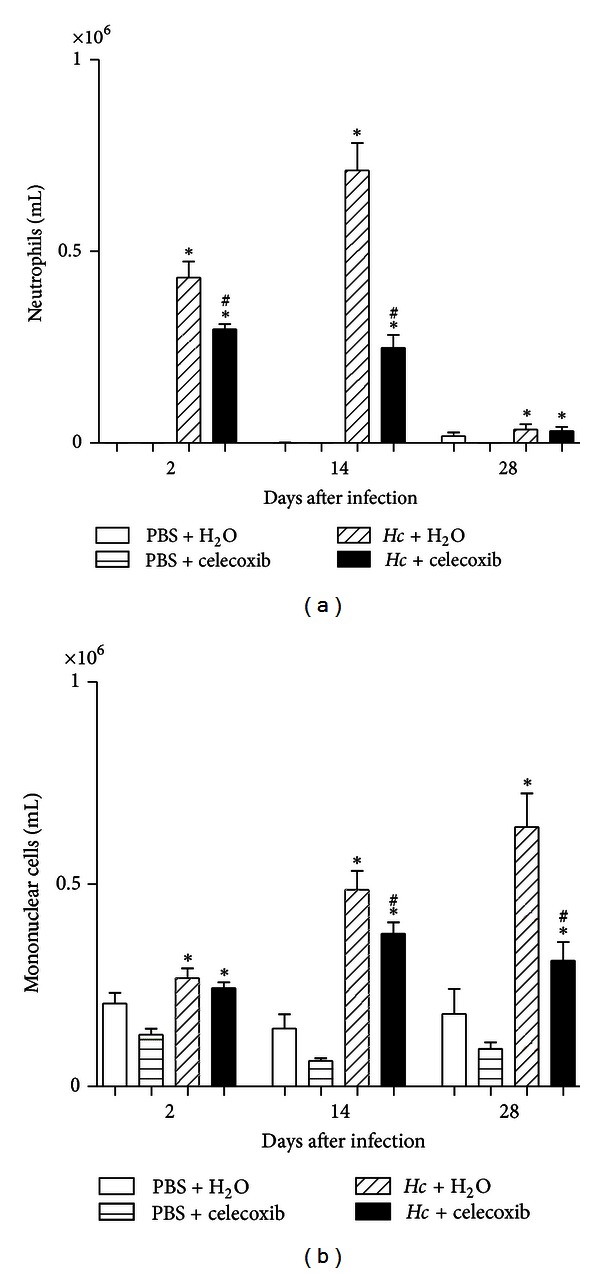
The inhibition of endogenous prostaglandin synthesis alters BALF leukocyte numbers during *H. capsulatum* infection. BALF cells were obtained from mice after i.t. injection of PBS or sublethal *H. capsulatum *infection and daily p.o. administration of water or celecoxib (1 mg/kg/0.5 mL). (a) Neutrophils and (b) mononuclear cells. The cells were enumerated and identified after cytospin preparation and panoptic staining. The data are presented as the means ± SEM representative of three independent experiments (*n* = 5-6). One-way ANOVA (Tukey's multiple comparison test) was used. *PBS + H_2_O versus *Hc* + H_2_O or versus *Hc* + celecoxib; ^#^ *Hc* + H_2_O versus *Hc* + celecoxib *P* < 0.05.

The influx of neutrophils and mononuclear cells into the bronchoalveolar space after the sublethal inoculation of *H. capsulatum *was significant at the onset of infection (day 2 after infection), peaked at 14 days after infection in neutrophils (Figure 4(a)), and increased in mononuclear cells after 28 days (Figure 4(b)) compared with noninfected mice. However, celecoxib treatment of infected mice significantly reduced the number of inflammatory cells in the BALF (Figures 4(a) and 4(b)), with the greatest inhibition of recruited neutrophils (~65%) and mononuclear cells (~49%) occurring at 14 and 28 days after infection, respectively. The treatment of noninfected mice with celecoxib did not modify the number of cells in the bronchoalveolar space at any of the time points examined. 

### 3.5. Immunophenotypic Characterization of Inflammatory Cells and Histology of Lung Parenchyma

**Figure 5:**
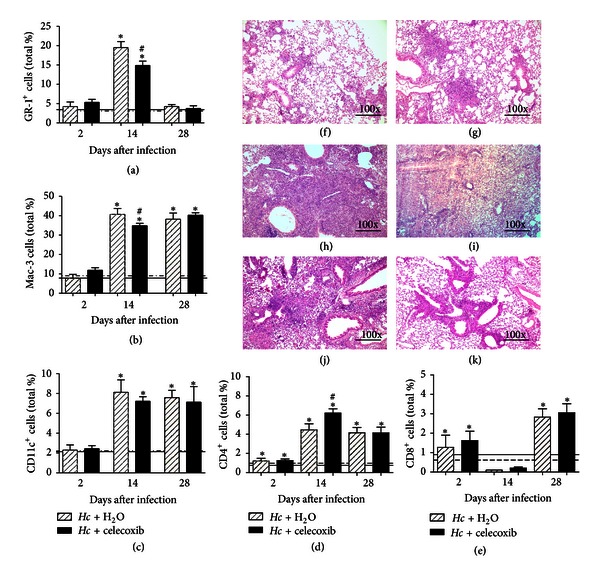
Celecoxib treatment reduces inflammatory cells in the lung parenchyma. At 2, 14, and 28 days after *H. capsulatum* infection, with or without celecoxib treatment, the phenotype of the cells present in the lung parenchyma was determined. The cells were marked with antibodies to GR-1 (a), MAC-3 (b), CD11c (c), CD4 (d), or CD8 (e). Myeloid and lymphoid populations were gated using forward/side scatters. A group of noninfected mice receiving water (solid line) and noninfected mice treated with celecoxib (dotted line) were used as controls. The lung tissues were also processed and stained with H & E to identify leukocyte infiltration after 2 (f, g), 14 (h, i), and 28 days (j, k). The lung sections from *H. capsulatum*-infected mice receiving water p.o. (f, h and j) or celecoxib (g, i and k). Magnification: 100x. The data are presented as the means ± SEM representative of three independent experiments (*n* = 5-6). One-way ANOVA (Tukey's multiple comparison test) was used. *PBS + H_2_O versus *Hc* + H_2_O or versus *Hc* + celecoxib; ^#^ *Hc* + H_2_O versus *Hc* + celecoxib *P* < 0.05.

The cells from the lung parenchyma of infected mice with or without celecoxib treatment were immunophenotyped at 2, 14, and 28 days after the sublethal infection with *H. capsulatum*. The GR-1 positive cells peaked at 14 days after infection and were similar to those of noninfected mice at 2 and 28 days after infection (Figure 5(a)). By contrast, at 28 days after infection, the persistence of MAC-3- and CD11c-positive cells was similar to that observed at 14 days (Figures 5(b) and 5(c)). Treating infected mice with celecoxib reduced the number of GR-1- (~24%) and MAC-3-positive cells (~15%) at 14 days after infection (Figures 5(a) and 5(b)). After celecoxib treatment, the percentage of CD11c- (Figure 5(c)) and CD8-positive cells (Figure 5(e)) was not altered, compared with those in infected mice during the entire observation period. Only the number of CD4-positive cells increased at 14 days after infection in mice treated with celecoxib compared with infected mice (Figure 5(d)). We also analyzed the infiltrating leukocytes in the lung parenchyma in histological sections. As expected, the lung recovered from untreated infected mice at 2 days after infection showed leukocyte infiltration, with a predominance of neutrophils and few macrophages with a substantial disorganization of the lung architecture (Figure 5(f)). At 14 days after infection, we observed diffuse granulomatous pneumonia and the severe destruction of lung parenchyma with the peribronchiolar and perivascular infiltration of macrophages and neutrophils (Figure 5(h)). After 28 days, we observed a predominance of macrophages and scant neutrophils in lung parenchyma and the moderate infiltration of inflammatory mononuclear cells in the peribronchiolar area (Figure 5(j)). Interestingly, in the lungs of mice infected and treated with celecoxib, the inflammatory process was restricted to a small accumulation of macrophages and neutrophils, with the preservation of alveoli and bronchi (Figures 5(g), 5(i), and 5(k)).

### 3.6. Celecoxib Treatment Alters the Production of Cytokines

**Figure 6:**
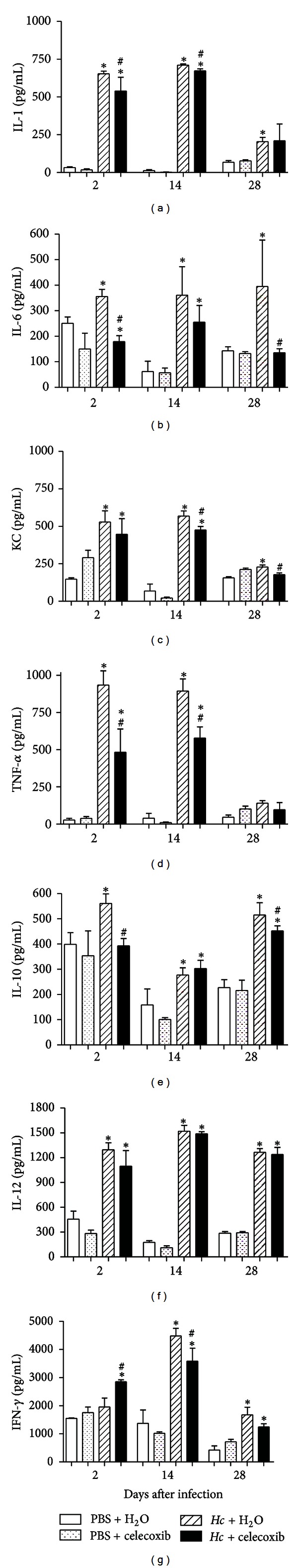
Treatment with celecoxib alters cytokine concentrations in the lung tissue of infected mice. The lungs were removed at 2, 14, and 28 days after i.t. inoculation and oral treatments and homogenized in medium. IL-1 (a), IL-6 (b), KC (c), TNF-*α* (d), IL-10 (e), IL-12 (f), and IFN-*γ* (g) concentrations were determined using ELISA. The data are presented as the means ± SEM representative of three independent experiments (*n* = 5-6). One-way ANOVA (Tukey's multiple comparison test) was used. *PBS + H_2_O versus *Hc* + H_2_O; ^#^ *Hc* + H_2_O versus *Hc* + celecoxib *P* < 0.05.

We also investigated whether celecoxib treatment altered cytokine production in the lungs of infected mice. As expected, the infection induced IL-1, IL-6, KC, TNF-*α*, IL-10, IL-12, and IFN-*γ* release at all time points compared with noninfected mice (Figure 6). The release of cytokines in the lung was altered after celecoxib treatment, varying according to the time point analyzed. At 2 days after infection, the levels of IL-1, IL-6, TNF-*α*, and IL-10 were reduced; at 14 days after infection, the levels of IL-1, KC, TNF-*α*, and IFN-*γ* were reduced, and a reduction in the IL-6, KC, and IL-10 concentrations was observed at 28 days after infection. Conversely, IFN-*γ* production increased at 2 days after infection, and cytokine production was not altered after treatment with celecoxib (Figure 6(g)) at 28 days.

### 3.7. Treatment with Celecoxib Increased Survival in Lethal *H. capsulatum* Infection

**Figure 7:**
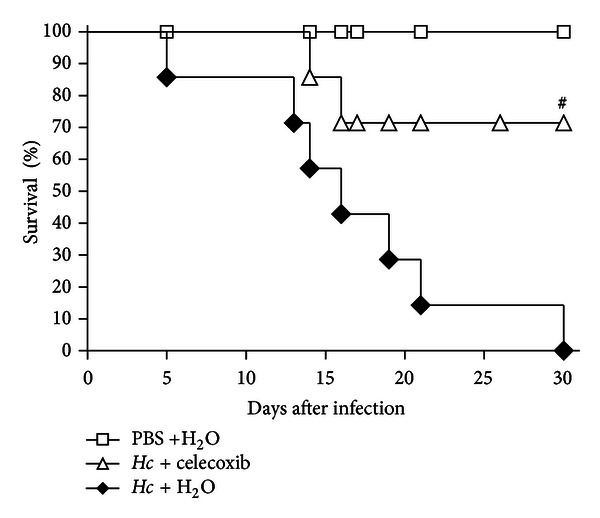
Celecoxib treatment increases the survival of mice infected with lethal inoculum of *H. capsulatum*. Mice infected with a lethal inoculum of *H. capsulatum* (1 × 10^6^ yeast) were treated daily p.o. with water or celecoxib (1 mg/kg/0.5 mL) for 30 days (*n* = 7). A group of noninfected mice were used as controls (*n* = 5). ^#^ *Hc* + H_2_O versus *Hc* + celecoxib. *P* < 0.05, using logrank test.

To assess the impact of prostaglandins on host defense against *H. capsulatum *infection, the mice were infected with a lethal *H. capsulatum *inoculum (1 × 10^6^ yeast/mice) and treated daily with celecoxib. The control infected mice were treated with vehicle. The animals were monitored for 30 consecutive days, and death was registered daily. Infected mice treated with 1 mg/kg of celecoxib showed an ~70% increase of survival compared with the water-treated infected mice (Figure 7). These data demonstrated that the inhibition of prostaglandins reduces the susceptibility of mice to *H. capsulatum *infection. Considering that prolonged treatment with celecoxib induces drug toxicity, we also infected mice with a sublethal inoculum of *H. capsulatum *(5 × 10^5^ yeast/mice) and administered daily treatments of celecoxib. We observed 100% survival after 60 days of treatment (data not shown), discharging the potential drug-induced toxicity.

## 4. Discussion

These studies of prostaglandin inhibition through celecoxib treatment during *Histoplasma capsulatum* infection revealed three principal new findings. First, the inhibition of COX-2 with celecoxib reduced the total fungal burden, the levels of PGE_2_ and cytokines, and the numbers of neutrophils and mononuclear cells in the bronchoalveolar space and lung parenchyma of infected mice. Second, celecoxib treatment increased the synthesis of nitric oxide, IFN-*γ*, and LTB_4_ and the phagocytic capacity of alveolar macrophages. Lastly, celecoxib treatment increased the survival of mice after infection with a lethal inoculum of *H. capsulatum*. 

The role of lipid mediators in the immune response to infectious agents has been increasingly recognized. These mediators participate in the cellular recruitment and modulation of effector mechanisms of the immune response, such as phagocytosis and killing and the generation and recruitment of memory T cells [8, 14–16, 18, 21–23]. Using celecoxib, we investigated the role of prostaglandins in pulmonary histoplasmosis and observed the increased phagocytosis of yeast by alveolar macrophages and the production of NO, IFN-*γ*, and LTB_4_ in the lung parenchyma after *H. capsulatum* infection. As the effector mechanisms of alveolar macrophages were enhanced, the survival was increased in *H. capsulatum*-infected mice, associated with reduced lung and spleen CFU. These results are consistent with the data obtained from other models of infection, demonstrating a pivotal role for prostaglandins in the pathogenic response to microbial infection [21, 22, 24–27]. Michelin et al. [21] employed a selective inhibitor of COX-2, salicylate or meloxicam, in a mouse model of *Paracoccidioides brasiliensis* and observed a reduction in the number of granulomas in the lungs and liver of infected mice. In another model, Michelin et al. [22] showed that *Trypanosoma-cruzi-*infected mice treated with sodium salicylate or meloxicam presented a significant reduction in blood parasitemia and mortality. In *Pseudomonas aeruginosa *pneumonia, the inhibition of COX-2 enhanced clearance [26], whereas the overexpression of COX-2 inhibited the clearance of bacteria [25]. Moreover, Serezani et al. [27] demonstrated that PGE_2_ inhibited the killing of *Klebsiella pneumoniae *and the production of reactive oxygen intermediates (ROI) by rat alveolar macrophages. Taken together, the results obtained in the present study and the data from the literature have shown that PGE_2_ possesses immunosuppressive effects in the immune response during different types of infections. We also investigated the mechanisms underlying the increase of mice survival after celecoxib treatment. In pulmonary histoplasmosis, neutrophils and mononuclear cells are recruited to the bronchoalveolar space during infection [8, 28]. In the present study, we demonstrated that the treatment with celecoxib increased CD4^+^, T cells, and reduced neutrophils, cells expressing the GR-1 marker, and the total number of mononuclear cells, including MAC-3- and CD11c-positive cells, in the bronchoalveolar space and lung parenchyma. Neutrophils and macrophages are fungistatic and fungicidal [29, 30], phagocytosing fungi and generating reactive oxygen, nitrogen intermediates, cytokines, and chemokines [31–34] in cooperation with dendritic cells [35] in response to pathogen infections. Considering that the celecoxib treatment reduced the production of inflammatory cytokines and chemokines and consequently the inflammatory reactions in the tissue (Figures 4, 5, and 6), it is reasonable to propose that celecoxib preserves the lung parenchyma and positively impacts the physiology of the lungs to increase the survival of infected mice.

Prostaglandins regulate cytokine production during *P. brasiliensis* infection [21]. As inflammatory cytokines are released during histoplasmosis [3, 4, 6–8], treatment with celecoxib could regulate this production. To address this issue, we measured the concentration of cytokines in the lung homogenates of *H. capsulatum*-infected mice with or without celecoxib treatment. We observed that the concentrations of IL-1, IL-6, KC, TNF-*α*, and IFN-*γ* (at 14 days) in the lung parenchyma were reduced after celecoxib treatment, showing that prostaglandins also regulate the release of these cytokines in *H. capsulatum* infection. Similarly, Kimura et al. [24] showed that NS-398 and nimesulide, two COX-2 selective inhibitors, inhibited PGE_2_ and IL-1, IL-6, KC, and TNF-*α* synthesis in neutrophils stimulated *in vitro* with zymosan. Furthermore, in *M. tuberculosis* infections, the oral administration of celecoxib reduced the concentrations of IL-1, IL-6, TNF-*α*, and IL-10 in the lungs [23]. IFN-*γ* and IL-12 play important roles in host protection against *H. capsulatum*, as exogenous IL-12 prevented mortality and induced IFN-*γ* release [7]. IFN-*γ* deficient mice have an altered balance of the cytokines critical for the resistance to histoplasmosis and present uncontrolled pulmonary infection [3]. Interestingly, we observed that prostaglandin inhibition reduced IL-10 and increased IFN-*γ* production at 2 days after infection and that IL-12 production was not altered at any time evaluated. Infected mice with or without celecoxib treatment presented the same number of CFU in the lungs at 2 days after infection, suggesting that the differences observed in cytokine production among the group were not due to the fungal load but indeed to the celecoxib treatment. Moreover, at 2 days after infection, the IFN-*γ* concentration was increased. Taken together, these results suggest that prostaglandins mediate IFN-*γ* production in the early phase and the release of other cytokines during lung infection. 

Resident alveolar macrophages are important cells that maintain lung sterility through the phagocytosis and killing of inhaled microorganisms in lung defense [36, 37]. The results obtained in the present study demonstrated that celecoxib treatment inhibited prostaglandin synthesis and increased the yeast phagocytosis, NO production, and killing activity of resident and inflammatory alveolar macrophages, potentially reflecting either the increased LTB_4_ and/or reduced PGE_2_ production. We previously demonstrated that the inhibition of leukotriene synthesis in infected mice reduced the antimicrobial host defense during *H. capsulatum* and *M.  tuberculosis* infections [8, 23, 38]. Leukotrienes improve the microbicidal activity of alveolar macrophages, increasing the production of NO [39], lysosomal enzymes, and defensins and the activation of NADPH oxidase [40, 41]. However, prostaglandins have an opposite effect on antimicrobial mechanisms compared with leukotrienes. PGE_2_ inhibited alveolar macrophage effector mechanisms, such as phagocytosis and pathogen killing via EP2 and EP4 receptors, through an increase in intracellular cyclic adenosine monophosphate (cAMP) [18, 19]. In addition, prostaglandins downregulate the expression of iNOS (inducible nitric oxide synthase) through the inhibition of NF-*κ*B production in lipopolysaccharide- (LPS-) stimulated J-774 macrophages [42]. Thus, the inhibition of prostaglandin synthesis contributes to increased phagocytosis and the killing of *H. capsulatum* by resident alveolar macrophages through a mechanism dependent on LTB_4_, NO, and IFN-*γ* production. Notably, the protection observed during the progression of infection cannot be exclusively associated with the inhibition of prostaglandins synthesis but is also due to a series of events, such as increased LTB_4_ and NO production in the lung parenchyma. NO is a potent killing mechanism for *H. capsulatum* [20, 43], and the roles of LTB_4_ and NO in the control of histoplasmosis infection have been previously demonstrated [8, 20, 43, 44]. The inhibition of prostaglandins increased the survival of mice infected with a lethal inoculum of *H. capsulatum* treated with celecoxib compared with infected animals, suggesting an important role for these lipid mediators during histoplasmosis. Notably, *H. capsulatum* releases lipid mediators, such as prostaglandins [45], and these mediators also reduce phagocytosis and killing by macrophages, thereby favoring fungal survival. Independent of the source of prostaglandins, these results suggest a harmful role for prostaglandins in murine pulmonary histoplasmosis. Thus, we suggest that the prostaglandins released from pulmonary cells during *H. capsulatum* infection contribute to the pathogenicity of this illness, and the inhibition of prostaglandins can be used as an adjuvant treatment for this mycosis.
